# Rare manifestations of pediatric chronic myeloid leukemia: a case report on priapism and a literature Review

**DOI:** 10.3389/fonc.2025.1579981

**Published:** 2025-06-19

**Authors:** Rawan Budair, Laith Baqain, Rawad Rihani

**Affiliations:** Department of Pediatrics, Blood and Marrow Transplantation and Cellular Therapy, King Hussein Cancer Center, Amman, Jordan

**Keywords:** ALL, acute lymphoblastic leukemia, B-ALL, B-cell acute lymphoblastic leukemia, CML, chronic myeloid leukemia, T-ALL, T-Cell acute lymphoblastic leukemia

## Abstract

Chronic myeloid leukemia (CML) is a myeloproliferative neoplasm characterized by uncontrolled myeloid cell proliferation and is primarily caused by a reciprocal chromosomal translocation [t(9;22)(q34;q11.2)]. Typical manifestations of CML include nonspecific constitutional symptoms such as fatigue, weight loss, night sweats, and abdominal discomfort due to hepatosplenomegaly. Although priapism is a rare but recognized complication of CML, it more often occurs in adults than in children. This case report describes an 11-year-old patient who experienced persistent priapism and hyperleukocytosis and ultimately received a CML diagnosis. Priapism in pediatric CML is a serious medical emergency requiring prompt medical and surgical intervention to prevent long-term complications, including the loss of erectile function. A literature review identified 19 pediatric cases of priapism associated with leukemia, 15 of which were attributed to CML. The cases varied in clinical presentation, treatment approaches, and outcomes, with management often involving a combination of aspiration, irrigation, leukapheresis, and chemotherapy. In most cases, priapism was resolved with these interventions, but some required additional measures, including shunt surgery. This review emphasizes the importance of early recognition and intervention to prevent complications in children with CML-associated priapism.

## Introduction

Chronic myeloid leukemia (CML) in childhood is uncommon, constituting 2–3% of leukemias in children under 15 years of age and 9% in adolescents 15–19 years of age. The hallmark of CML is the Philadelphia chromosome, which results from a translocation between chromosomes 9 and 22 (1). The BCR-ABL1 oncoprotein, which causes clonal expansion of affected hematopoietic cells, is central to the pathogenesis of CML (2). Children, especially in the chronic phase of CML, typically present with hyperleukocytosis, splenomegaly, and other general symptoms such as fatigue, weight loss, night sweats, left upper quadrant abdominal discomfort, and thrombosis or bleeding events (3). Priapism is a urological emergency defined as a prolonged pe erection persisting beyond 4 hours, irrespective of sexual arousal. This condition can arise from an imbalance in penile blood dynamics and is categorized as low-flow (ischemic) or high-flow (non-ischemic) (4). CML-associated priapism, particularly in pediatric patients, is rare but primarily ischemic due to a hyperleukocytosis-associated increase in blood viscosity (5).

## Case report

A previously healthy 11-year-old male patient entered the emergency room with a painful, 3-day penile erection that was neither sexual nor trauma-induced. No similar episodes were encountered previously. He also experienced fever and night sweats for 1 week before hospital admission.

**Figure 1:**
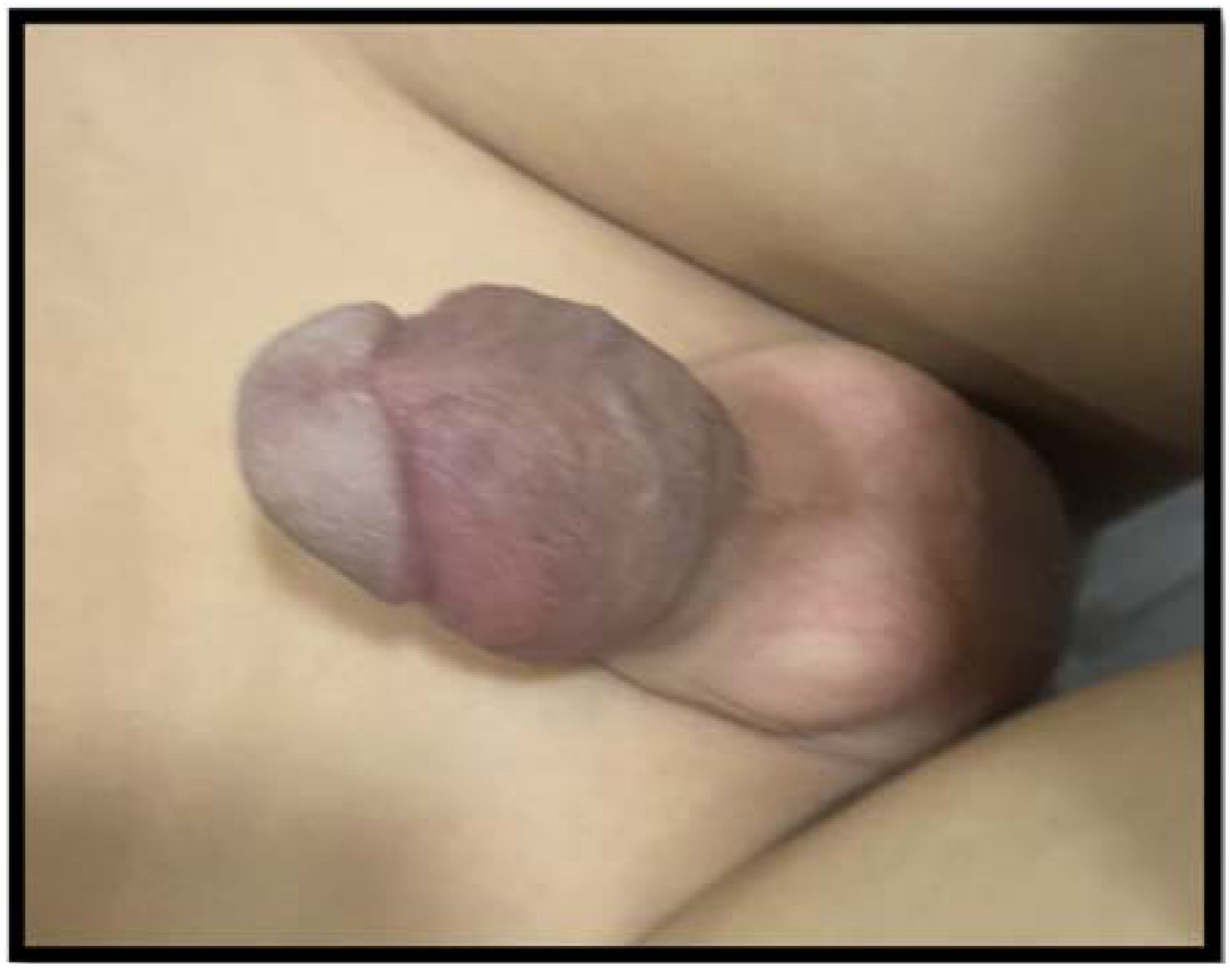
Priapisim on initial presentation.

During the initial encounter in the emergency room, the patient was pale and in severe pain. A physical examination identified pallor and a severely swollen, erythematous, erect, and tender to touch penis (**Figure 1**). There were no signs of infection of the overlying scrotal or penile skin, and there was no abnormal urethral discharge or inguinal lymph node enlargement. The patient also had massive splenomegaly that reached the umbilicus.

Laboratory tests showed a hemoglobin level of 8.9 g/dL, a white blood cell (WBC) count of 360 × 103/µL (neutrophils 90.3%, lymphocytes 1.7%, basophils 1.9%, eosinophils 2.9%, platelets 932 × 103/µL), and lactate dehydrogenase of 1294 U/L. A blood film analysis identified leukocytosis with absolute basophilia, and serum chemistry was unremarkable.

**Figure 2:**
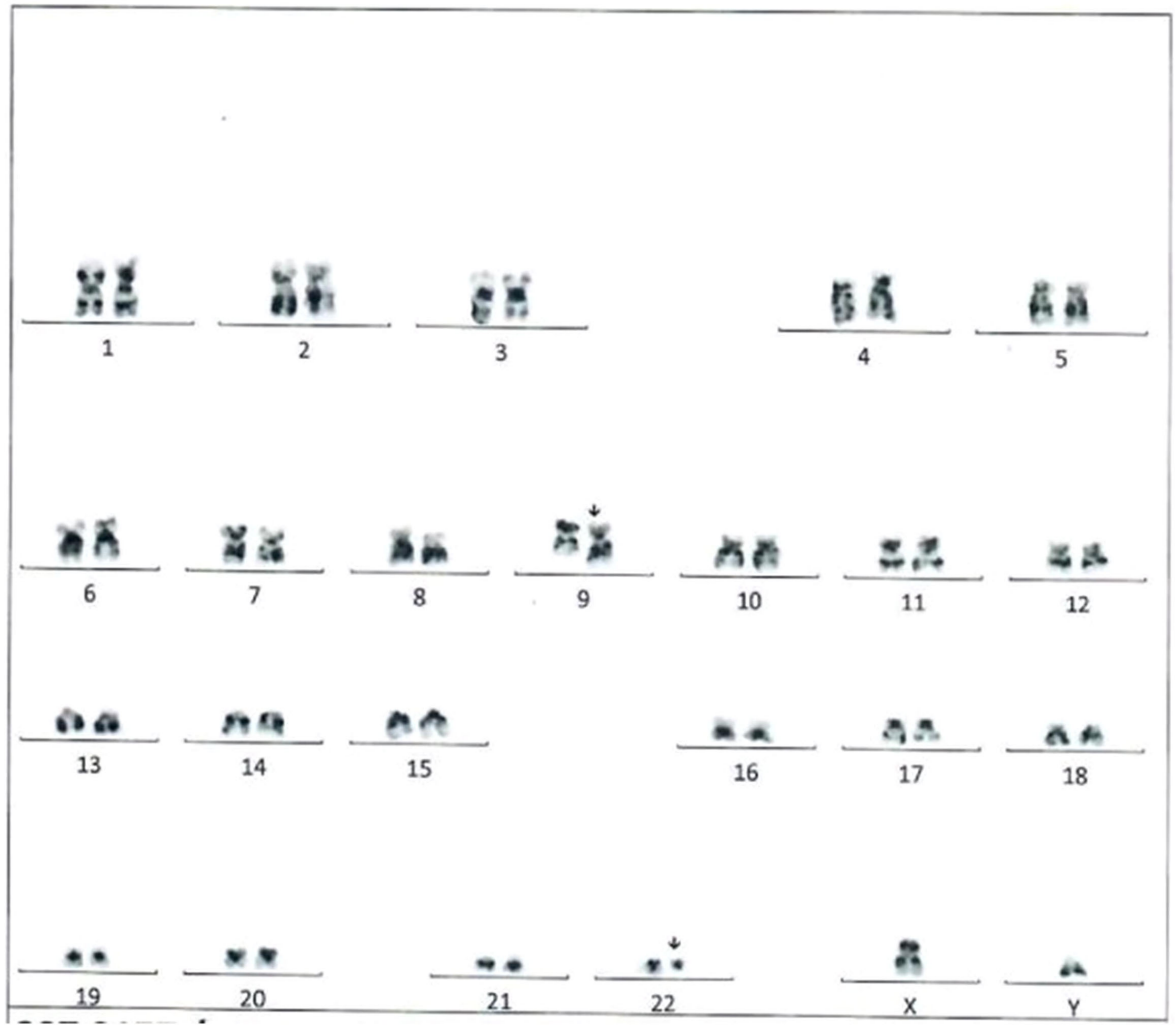
Karyotype analysis in the reported patient indicating the translocation t(9,22){q34;q11.2}.

The patient was admitted to the pediatric intensive care unit for further evaluation, symptom management, and observation. He received vigorous hydration, morphine sulfate continuous infusion, and IV antibiotics. He also received 20 mg/kg hydroxycarbamide and frequent monitoring of laboratory features. Cytogenetics showed that 94% of peripheral blood cells were positive for the BCR-ABL1 t(9;22)(q34;q11.2) rearrangement (**Figure 2**). Fluorescence *in situ* hybridization also identified the BCR-ABL1 t(9;22)(q34;q11.2) rearrangement. Flow cytometry analysis identified myeloblasts that were positive for CD34, CD117, and HLA-DR.

On the second day of treatment, the patient’s WBC count dropped to 293 × 103/µL and he started receiving imatinib mesylate (340 mg/m2/day). The urology team was involved early during therapy, but their evaluation was consistent with corpora cavernosal tissue damage due to the duration of symptoms. Insertion of a 23-gauge butterfly needle at 3 and 9 o’clock enabled the aspiration of 3–4 ml of clotted blood, followed by irrigation with normal saline. Further aspiration resulted in partial detumescence. The patient received an intracorporal injection of 15 µg of adrenaline to decrease pain and relieve the erection, after which the pain slightly improved but the penis remained rigid. Penile blood gas measurements showed severe acidosis.

The care team conducted continuous monitoring and frequent clinical examinations to assess clinical response and determine the next steps in surgical management, such as possible distal shunting if the erection persisted. The patient received enoxaparin sodium and was treated with leukapheresis. The next day, he developed external edema at the aspiration site, but the erection was softer. Foley’s catheter was inserted to bypass any possible obstruction or urine retention.

The leukocyte count after leukapheresis was 278 × 103/µl, so the patient underwent a second leukapheresis session. His leukocyte count remained high after the second session (242 × 103/µL), so the patient received low-dose cytarabine as cytoreductive (100 mg/m2 twice daily for 4 days). Meanwhile, conservative management was continued, including cold compresses at the root of the penis and pain control by continuous infusion of morphine sulfate and oral administration of gabapentin. No further surgical interventions were needed.

After the administration of low-dose cytarabine, the patient’s leukocyte count decreased gradually to 120 × 103/µl. He continued to have a swollen penis, but the erection and rigidity improved substantially with the combination of cytarabine, imatinib mesylate, and hydroxyurea, along with cold compresses.

## Discussion

In this report, we present a rare case of priapism in a pediatric patient with CML. The marked leukocytosis and significant splenomegaly indicated CML, which caused priapism. This report also reviews previous cases of priapism in pediatric patients (under 18 years of age) with CML (**Table 1**).

**Table 1:** Characteristics , features and management of patients with priapism in CML.

Year	Authors	Age (years)	Diagnosis	Priapism Onset Duration till presentation to hospital	Priapism Type	Other Symptoms	Initial WBC×10⁹/L	Initial Platelet Count ×10⁹/L	Initial Hb g/dL	Splenomegaly	Treatment with Hydroxyurea	Leukapheresis	Aspiration and Irrigation	Shunt	Other Chemotherapy/Medical Therapy	Duration of Priapism Till Resolution (Days)	Prognosis/ Outcome
1971	Leonard Jaivier et al. (6)	7 weeks	CML with blast crisis	5 days	Ischemic	Bilateral inguinal lymph nodes enlargement	37	344	10	Yes	No	No	No	Yes	NA	NA	Recovery
2003	Hans-Joachim Mentzel et al. (7)	12	ALL	NA	High flow	NA	618	NA	NA	No	No	Yes	No	No	Prednisone	5	Recovery
2004	Rebecca Werther et al. (8)	3	T-ALL	3 hours	Ischemic	NA	606	20	7	Yes	No	Yes	No	No	Prednisone VCR, DNR, Asparginase	8	Recovery
2007	Gupta, Seth et al. (9)	12	CML	2 days	Ischemic	Pallor and fatigue	346	924	9	Yes	Yes	No	No	No	Imatinib Mesylate, Terbutaline	3	Recovery
2011	Narendra et al. (10)	11	CML	12 hour	Ischemic	NA	290	550	7.3	Yes	Yes	No	No	No	Imatinib Mesylate	NA	Recovery
2012	Veljkovic et al. (11)	16	CML	1 day	Ischemic	NA	320	417	11	Yes	No	Yes	Yes	No	NA	13	Recovery
2013	Shankar Prasad Hazra et al. (12)	14	CML	1 day	Ischemic	pallor	226	310	9.9	Yes	Yes	No	Yes	No	NA	6	Recovery
2015	Ergenc et al. (13)	18	CML	72 hour	Ischemic	NA	100	1002	6	Yes	No	Yes	No	No	Imatinib Mesylate	NA	Recovery
2017	Musa et al. (14)	18	CML	12 days	Ischemic	NA	199	504	5.7	Yes	Yes	No	No	No	NA	28	Recovery
2017	Minckler et al. (15)	18	CML	6 hours	Ischemic	NA	588	109	7.3	Yes	Yes	No	Yes	No	NA	NA	Recovery
2018	Qu, Lu et al. (16)	18	CML	7 days	Ischemic	Pallor	257	5450	7.1	Yes	No	No	Yes	Yes	Imatinib Mesylate	NA	Recovery
2018	Khan, Shafiq et al. (17)	16	CML with blast crisis	11 days	Ischemic	Pallor	614	907	5.7	Yes	Yes	No	Yes	Yes	NA	NA	NA
2018	Atas et al. (18)	18	CML	5 days	Ischemic	NA	215	NA	NA	No	No	Yes	No	No	NA	NA	Recovery
2018	Clark et al. (19)	13	CML	3 days	Ischemic	NA	350	450	8.5	Yes	Yes	Yes	Yes	Yes	Imatinib Mesylate	NA	Recovery
2018	Gupta et al. (20)	10	B-ALL	2 days	Ischemic	Fever , pallor , headache	693	40	5	No	No	No	Yes	No	Dexamethasone	4	Died 2 days from admission
2019	Avtar Singh Dhanju et al. (21)	18	CML	14 hours	Ischemic	Fever , fatigue	363	527	9.7	Yes	No	No	Yes	No	Imatinib Mesylate	1	Recovery
2019	Purnima Thakur et al. (22)	15	CML	2 days	Ischemic	Epistaxis , weight loss	135	197	9	Yes	No	No	Yes	Yes	Imatinib Mesylate	4	Recovery
2020	Oumar Gaye et al. (23)	9	AML	36 hour	Ischemic	Pallor,gingival hypertrophy	82	810	3.4	Yes	No	No	Yes	No	VCR/Prednisolone	2	Recovery
2021	Siprianus Ugroseno Yudho Bintor et al. (24)	18	CML	20 day	Ischemic	Tinnitus, blurred vision	421	407	10.4	Yes	Yes	Yes	No	No	Imatinib Mesylate	32	Recovery

CML is a recognized cause of ischemic priapism, primarily due to hyperleukocytosis and the resulting leukocyte aggregation within the sinusoids of the corpora cavernosa. This process causes sinusoidal engorgement and an erection that obstructs venous outflow through the emissary veins (25). An enlarged spleen may also mechanically compress abdominal veins, further contributing to venous congestion in the corpora cavernosa (26).

Hematologic disorders, particularly sickle cell anemia and leukemia, are the predominant causes of priapism in pediatric patients (26). Leukemic priapism is typically of the low-flow, ischemic type triggered by hyperleukocytosis (12). Although CML often presents with nonspecific symptoms, priapism is an exceptionally rare initial manifestation in pediatric cases. Given the rarity of cases, medical professionals have limited clinical experience with pediatric CML involving priapism.

Common features of CML, including leukocytosis, often lead to complications such as pulmonary leukostasis, which can result in acute respiratory distress (27). Additional symptoms may include neurological manifestations such as headache, dizziness, and visual or auditory disturbances (28, 29).

Few reports describe priapism as an initial presentation of pediatric CML (30), but some describe its occurrence during therapy for CML (1, 26, 28). Most pediatric patients with CML involving priapism initially had a median hemoglobin of 11.1 g/dL, a median WBC count of 250 × 103/µL (range, 8 × 103–800 × 103/µL), and a mean platelet count of 500 × 103/µL (range, 40 × 103–2 × 106/µL), along with notable splenomegaly.

Among cases reviewed in the literature, priapism in pediatric CML was observed at a median age of 13 years (range, 9–53), with an average duration of 36 hours (range, 18 hours to 7 days) (5).

Priapism is a urological emergency that may have a poor prognosis, with a 50% risk of impotence despite appropriate management (30). Reports consistently emphasize the emergent nature of this condition in hematologic disease (26, 30), highlighting the need for prompt and accurate diagnosis. This underscores the importance of thorough history -taking, clinical examination, laboratory tests, and imaging studies (24).

Management of priapism typically involves a combination of aggressive supportive care and systemic treatments (26). As ischemic priapism is a compartment syndrome, early interventions such as hydration, pain relief, cytoreductive therapies, and leukapheresis are crucial. Pain associated with priapism can be severe and may not respond to high-dose analgesics. In cases of refractory pain, leukapheresis can alleviate symptoms by rapidly reducing leukocyte counts (30).

Several case series have reported successful outcomes with therapeutic leukapheresis for priapism (30), often in conjunction with cytotoxic therapy (11). Commonly used local interventions include the aspiration of blood from the corpora cavernosa followed by saline irrigation to clear sludged blood (31). If these measures are ineffective, a vasoconstrictive agent such as phenylephrine (100–200 µg/mL) can be administered in 5-minute intervals until complete detumescence is achieved. In resistant cases, surgical shunting may be considered, as recommended by the American Urological Association. This procedure creates a shunt between the corpus cavernosum and the glans penis, corpus spongiosum, or a vein to bypass the obstructed veno-occlusive mechanism (31) (“How I manage priapism in chronic myeloid leukaemia patients - PubMed,” n.d.).

Few case reports specifically describe the management of pediatric priapism associated with CML (24). Some groups reported the resolution of priapism after aspiration, irrigation, or shunt surgery, whereas others noted successful outcomes with CML-specific treatments, including leukapheresis and chemotherapy (24).

In eight cases of CML-associated priapism, complete resolution was achieved by using priapism-specific interventions, including either aspiration and irrigation or shunting. Shankar and Hazra (12) reported successful resolution of priapism in a 14-year-old male who presented with pallor, leukocytosis (WBC 226 × 103/µL), and a 1-day history of priapism. Aspiration and irrigation resolved the priapism after 6 days. Similarly, Khan et al. (17) described a 16-year-old male with CML in blast crisis and an initial WBC of 614 × 10⁹/L, platelet count of 907 × 10⁹/L, and hemoglobin level of 5.7 g/dL. His priapism resolved within 2 days after aspiration, irrigation, and shunt surgery.

Graiver et al. (6) reported a unique case of infantile priapism in a 7-week-old with CML in blast crisis and an initial WBC of 37 × 10⁹/L. This patient required both aspiration and irrigation and shunt surgery. Minckler et al. (15) described an 18-year-old male with an initial WBC of 588 × 10⁹/L whose priapism was resolved through aspiration and irrigation approximately 1 day after presentation.

Thakur et al. (22) reported on a 15-year-old with CML who had a 2-day history of painful priapism, epistaxis, and weight loss. With aspiration, irrigation, and shunt surgery, the patient achieved detumescence 4 days after priapism onset.

Nerdana et al. (10) described an 11-year-old male with CML and an initial WBC of 290 × 10⁹/L whose priapism was resolved through supportive measures. Avtar et al. (21) described an 18-year-old who had 14 hours of priapism, generalized fatigue, fever, and a WBC count of 363 × 10⁹/L. After aspiration and irrigation, his priapism resolved 1 day after initiation of CML-specific therapy.

Qu Lu et al. (16) described an 18-year-old male presenting with priapism and pallor lasting 7 days, with a hemoglobin level of 7.1 g/dL, platelet count of 5450 × 10⁹/L, and a WBC count of 257 × 10⁹/L. Initial aspiration and irrigation did not resolve the priapism, so shunt surgery was required.

We identified seven pediatric cases requiring leukapheresis for priapism management, with some requiring multiple sessions (range, 2 to 8) as did the patient in our study. Werther et al. (8) reported a 3-year-old with acute T-cell lymphoblastic leukemia who presented with a 3-hour history of painful priapism and a WBC count of 606 × 10⁹/L. This case required leukapheresis and chemotherapy, resulting in detumescence after 8 days of treatment. In the reported cases, leukapheresis effectively reduced blast cell numbers but was insufficient on its own due to the risk of blast cell rebound, making systemic treatment essential to address the underlying condition (32).

After priapism resolution, patients should receive leukemia- or CML-specific therapies, including tyrosine kinase inhibitors (TKIs) (3, 32). Pediatric patients with confirmed CML and priapism at initial presentation require a combination of medical therapies such as hydroxycarbamide, prednisolone, dexamethasone, vincristine, TKIs, cytarabine, and anticoagulants (28), along with surgical interventions to achieve priapism resolution (1).

Reversing priapism through medical therapy generally takes longer than other methods such as aspiration, leukapheresis, irrigation, and shunting (26). Previous studies show that priapism in CML presents with variable clinical manifestations, duration, and treatment responses (31). Management strategies are diverse and depend on the initial presentation and clinical response, with all approaches aiming for complete resolution of priapism and control of the underlying disease.

In adults, priapism is also uncommon but occurs more frequently than in children, with a bimodal incidence peak between ages 5 and 10 in children and 20 and 50 in adults (3). Adults may also present with symptoms such as unprovoked priapism, abdominal pain, bruising, bleeding, and weakness. The standard first-line treatments for acute ischemic priapism, which involve surgical blood drainage, are often unsuccessful in preserving erectile function in adults (31).

This report has several limitations, including the limited number of reported pediatric patients with priapism, which restricts the generalizability of our findings. Additionally, the long-term assessment of sexual function in survivors was not feasible.

This review emphasizes that early clinical recognition and intervention are key to the successful treatment and prevention of late complications of priapism. Systemic intervention combined with local therapy improves the likelihood of successful outcomes.

## Conclusion

Although priapism rarely occurs during the initial presentation of CML in children, this condition requires immediate medical and surgical attention. Early interventions targeting the underlying cause, coupled with localized treatment, are essential to reduce the risk of long-term sequelae such as erectile dysfunction.

## Data Availability

All relevant data is contained within the article: The original contributions presented in the study are included in the article/supplementary material, further inquiries can be directed to the corresponding author/s.
